# Advancing Wheat Productivity Through Nutrient Interactions, Fertilizer Practices, and Genetic Improvement

**DOI:** 10.3390/life16050795

**Published:** 2026-05-10

**Authors:** Ibrahim S. Elbasyoni, Soleman M. Al-Otayk, Mohamed Ghonimy, Mohamad I. Motawei

**Affiliations:** 1 Plant Science Program, Biological and Environmental Science and Engineering Division, King Abdullah University of Science and Technology (KAUST), Thuwal 23955, Saudi Arabia; ibrahim.elbasyoni@kaust.edu.sa; 2 Crop Science Department, Damanhur University, Damanhur 22516, Egypt; 3 Department of Plant Production, College of Agriculture and Food, Qassim University, Buraydah 51452, Saudi Arabia; satiek@qu.edu.sa; 4 Department of Agricultural and Biosystems Engineering, College of Agriculture and Food, Qassim University, Buraydah 51452, Saudi Arabia

**Keywords:** wheat, plant, nutrient interactions, fertilization strategies, biofortification, zinc, Selenium, genetic improvement, nutrient-use efficiency

## Abstract

Wheat (*Triticum aestivum* L.) is a cornerstone of global nutrition yet yield-focused intensification has often overlooked the biological complexity of nutrient interactions and their implications for nutritional outcomes. This review synthesizes current advances in wheat nutrient management from a systems perspective, integrating nutrient interactions, fertilization practices, and genetic improvement. A key novelty of this work is the development of a conceptual framework that links nutrient interaction networks with genotype-specific and environment-dependent responses, providing a unified approach to optimizing wheat productivity. Evidence indicates that plant performance is governed by coordinated nutrient dynamics rather than isolated inputs, with interactions such as nitrogen and sulfur playing a central role in regulating nutrient-use efficiency and metabolic processes. In addition, targeted micronutrient management, particularly zinc and selenium, is highlighted as a practical pathway for agronomic biofortification and enhanced nutritional value. The review further emphasizes substantial genetic variation in nutrient-use efficiency and yield stability, supporting the integration of breeding strategies with fertilization approaches. Emerging tools, including genomic-assisted selection and gene editing, are discussed as enabling technologies. Overall, this synthesis advances a biologically informed framework for sustainable wheat production that improves yield and nutritional outcomes.

## 1. Introduction

Wheat (*Triticum aestivum* L.) is one of the most widely cultivated cereal crops worldwide and represents a cornerstone of global food security, providing nearly one-fifth of the calories and protein consumed by humans [1]. In addition to its caloric contribution, wheat grain serves as an important dietary source of essential micronutrients, including zinc (Zn), iron (Fe), selenium (Se), and magnesium (Mg), which are vital for human metabolism, immune function, and overall health [2]. Productivity, improving wheat productivity while maintaining or enhancing its nutritional value, has become a central objective in efforts to achieve sustainable food and nutrition security.

Over recent decades, wheat production has increased substantially due to the widespread adoption of high-yielding cultivars, expansion of irrigation systems, and intensified fertilization practices associated with the Green Revolution [3]. While these advances have significantly improved global grain production, they have also led to unintended consequences, particularly with respect to grain nutritional composition. The phenomenon of nutrient dilution, whereby increasing yield is often associated with reduced concentrations of essential micronutrients, has been widely reported in wheat and other cereal crops [2,4]. As a result, modern high-yielding cultivars may exhibit lower micronutrient densities compared with traditional varieties, raising concerns regarding the long-term nutritional quality of staple foods.

At the same time, global agricultural systems are increasingly challenged by climate change, soil degradation, declining soil fertility, and inefficient nutrient management practices. Imbalanced fertilizer use has contributed to nutrient losses, soil acidification or alkalization, reduced soil organic matter, and declining nutrient-use efficiency in many cropping systems [5]. These challenges not only threaten agricultural sustainability but also increase production costs and environmental risks. Therefore, optimizing fertilization practices to improve nutrient efficiency while maintaining soil health has become a critical priority.

Recent advances in plant nutrition research emphasize that crop performance is not determined solely by the availability of individual nutrients but rather by complex interactions among macro- and micronutrients within the soil–plant system. Nutrient synergism and antagonism can strongly influence nutrient uptake, translocation, and metabolic processes, ultimately affecting plant growth and productivity [6,7,8]. For example, interactions between nitrogen (N) and sulfur (S) play a fundamental role in regulating nitrogen-use efficiency (NUE), protein synthesis, and key metabolic pathways in wheat [5,8]. Similarly, interactions between nitrogen and micronutrients such as zinc can enhance nutrient uptake efficiency, influence enzymatic activity, and affect grain nutritional composition [5]. These findings highlight the importance of understanding nutrient interactions as a basis for developing more effective fertilization strategies.

In this context, integrated approaches to nutrient management, including Integrated Nutrient Management (INM), have been proposed as effective strategies for optimizing nutrient availability and improving soil fertility. These approaches combine mineral fertilizers, organic amendments such as farmyard manure and compost, and biological inputs including microbial biofertilizers to enhance nutrient cycling and soil biological activity [6]. Such strategies have been shown to improve wheat growth, yield components, and long-term soil productivity [6,7]. However, their effectiveness depends on the interactions among nutrients, plant genotypes, and environmental conditions, highlighting the need for a more systems-oriented perspective that integrates fertilization practices with plant biological responses.

Improving the nutritional quality of wheat has also gained increasing attention through the concept of biofortification. Biofortification involves enhancing the concentration of essential micronutrients in edible plant parts through agronomic practices, conventional breeding, or biotechnological approaches [9]. Agronomic biofortification, particularly through the application of micronutrients such as zinc and selenium, has demonstrated significant potential for increasing grain micronutrient density and addressing hidden hunger, a widespread form of malnutrition affecting large populations globally [2]. However, the effectiveness of agronomic interventions may vary across environments and production systems.

Genetic approaches provide a complementary and more sustainable pathway for improving wheat nutritional quality [10]. Conventional breeding aims to enhance nutrient uptake, translocation, and accumulation in grains, although the quantitative inheritance of traits such as grain Zn and Fe concentration can limit progress [11,12]. To overcome these constraints, advanced strategies including marker-assisted selection and genomic selection are increasingly used to accelerate genetic gains [13]. In addition, transgenic approaches and gene-editing technologies, such as CRISPR-based methods, enable targeted modifications of genes involved in nutrient transport and storage [14]. For example, genes such as *Gpc-B1* have been associated with increased protein content and enhanced micronutrient accumulation, although potential trade-offs with yield may occur [15,16]. Furthermore, biotechnological interventions targeting carotenoid biosynthesis, mineral bioavailability, and anti-nutritional factors have demonstrated additional opportunities for improving wheat nutritional value [15,17,18,19].

Relevant literature was collected from peer-reviewed journal articles addressing nutrient effects in wheat, with particular emphasis on studies reporting yield, grain quality, and nutrient interaction responses under different environmental and management conditions. Studies were selected based on their scientific relevance, clarity of experimental description, and availability of quantitative or interpretable results. Both field and controlled-environment studies were included to capture agronomic responses under realistic production conditions as well as physiological and mechanistic insights under controlled settings. In addition, modeling and simulation studies were considered where they contributed to understanding nutrient dynamics and system-level responses. For analytical clarity, the selected studies were grouped into three categories according to experimental design: (i) field-based agronomic trials conducted under real production environments, (ii) controlled-environment or greenhouse experiments, and (iii) modeling or simulation studies. Key response variables, including grain yield and grain quality traits (e.g., protein content), were extracted from the selected studies. Greater interpretative weight was given to field-based studies due to their direct relevance to agronomic practice, while controlled and modeling studies were primarily used to support mechanistic explanation and broader system understanding. This approach provides a transparent and structured narrative synthesis while maintaining flexibility in integrating diverse types of evidence.

Nutrient management outcomes are strongly influenced by agroecological conditions, including soil characteristics, climate variability, cropping systems, and wheat genotype. In arid and semi-arid environments, challenges such as low soil organic matter, limited water availability, and reduced micronutrient availability can significantly constrain nutrient-use efficiency. Under such conditions, balanced fertilization strategies combined with organic amendments and biological inputs can enhance soil fertility and crop performance [5].

To improve the geographical representativeness of the synthesis, the revised analysis incorporates evidence from a broad range of agroecological regions, including Europe, North America, South Asia, and Australia, covering both temperate and semi-arid production systems. While earlier studies were largely concentrated in calcareous soils of arid regions such as Saudi Arabia where sulfur deficiency is frequently reported due to low organic matter and limited sulfate availability, the updated synthesis integrates findings from diverse soil types and cropping systems. Recent meta-analytical and field-based studies consistently demonstrate that sulfur fertilization enhances wheat yield and grain protein content across environments, although the magnitude of response is strongly influenced by soil properties, climate, and nitrogen management practices [20,21,22,23,24]. These effects are linked to improved nitrogen-use efficiency and enhanced sulfur–nitrogen metabolic coordination, which are physiologically conserved across cropping systems [20]. Overall, this expanded synthesis reduces regional bias while strengthening the global applicability of conclusions on nitrogen–sulfur interactions in wheat. Importantly, interactions among genotype, environment, and management practices (G × E × M) play a crucial role in determining yield stability and nutrient accumulation, emphasizing the importance of context-specific and genotype-responsive approaches.

Given these challenges and opportunities, a comprehensive understanding of nutrient interactions, fertilization practices, and genetic improvement is essential for advancing wheat productivity under diverse production systems. Integrating these components provides a systems-oriented framework for optimizing nutrient-use efficiency, enhancing plant performance, and improving nutritional outcomes. Thus, this review aims to examine the role of INM in wheat growth, yield performance, and grain quality; analyze synergistic interactions among macro- and micronutrients and their effects on nutrient uptake and plant physiology; evaluate agronomic biofortification strategies for key micronutrients—particularly zinc, selenium, and magnesium; compare genotype-specific responses across organic and conventional systems; and outline future perspectives for developing nutrient-efficient and climate-resilient wheat production systems through integrated management and advanced breeding approaches.

## 2. Method of Review

This review synthesizes recent experimental, field, and meta-analysis studies that investigate the role of INM in improving wheat productivity, grain quality, and micronutrient enrichment under diverse agroecological conditions. It should be noted that this is a narrative literature review, rather than a formal systematic review; nevertheless, all steps were carefully structured to ensure transparency, clarity, and reproducibility to the extent possible. Emphasis was placed on studies examining nutrient interactions, agronomic biofortification strategies, and nutrient management practices designed to enhance yield stability and nutritional value in wheat production systems.

The literature was compiled using several major scientific databases, including Scopus, Web of Science, ScienceDirect, SpringerLink, and Google Scholar. Priority was given to peer-reviewed journal articles published between 2005 and 2025, although earlier seminal publications were included when necessary to clarify fundamental concepts related to plant nutrition, nutrient interactions, and biofortification in cereal crops. Both experimental and review articles were considered when they provided relevant insights into wheat nutrient management and grain nutritional enhancement.

The literature search combined controlled vocabulary terms with broader keywords to capture relevant studies across agronomy, soil science, and plant nutrition disciplines. Key search terms included INM, wheat fertilization, nutrient interactions, nitrogen–sulfur interaction, zinc fertilization, agronomic biofortification, micronutrient enrichment, grain protein accumulation, soil fertility management, nutrient-use efficiency, and wheat yield and quality. Boolean operators were used to refine the search strategy and identify studies addressing both nutrient management practices and wheat nutritional outcomes. The final body of evidence synthesized in this review is based on 117 peer-reviewed publications, ensuring adequate coverage of nutrient interactions, fertilization strategies, and wheat productivity responses.

Studies were selected when they provided experimental or field-based evidence evaluating nutrient management practices in wheat, examined interactions among macro- and micronutrients affecting wheat growth and grain quality, investigated agronomic biofortification strategies aimed at increasing micronutrient concentrations in wheat grain, or analyzed nutrient-use efficiency and soil fertility dynamics under integrated fertilization systems. Studies lacking quantitative data, focusing exclusively on non-cereal crops, or addressing unrelated aspects of crop management were excluded from consideration.

Although this review adopts a narrative synthesis approach, consideration was given to the methodological robustness of the included studies to enhance the transparency of evidence interpretation. In this context, studies were appraised based on three main aspects: experimental setting, with field-based studies generally providing higher ecological validity compared to greenhouse experiments; sample size, referring to the number of experimental units or observations reported within each study; and experimental replication, reflecting the number of independent repetitions used to ensure reliability of results. These aspects were not applied as exclusion criteria but were used to inform the comparative interpretation of findings during synthesis, whereby studies with more rigorous experimental designs and stronger statistical reliability were given greater interpretative consideration.

The selected literature was organized into five major thematic groups to facilitate structured discussion and interpretation. These groups include:

1. Concepts and components of integrated nutrient management in wheat production systems.
2. Synergistic and antagonistic interactions among macro- and micronutrients influencing wheat growth and grain quality.
3. Agronomic biofortification strategies for enhancing micronutrient concentrations in wheat grain.
4. Effects of integrated nutrient management on wheat yield performance and grain nutritional composition.
5. System-specific nutrient management strategies for improving nutrient efficiency and sustainability in wheat-based cropping systems.

These thematic groups align with the structured sections and subsections of the manuscript, ensuring a coherent and logical presentation of evidence throughout the review.

The synthesis approach relied on thematic and integrative analysis to identify consistent trends, knowledge gaps, and emerging research directions across literature. Patterns in nutrient interactions, biofortification efficiency, and yield responses were critically compared across different soil types, climatic conditions, and wheat genotypes, providing a comprehensive and balanced interpretation of the available evidence.

It should be acknowledged that the included studies exhibit methodological diversity in terms of experimental design, scale, and replication intensity, which is inherent in agronomic research conducted under different environmental conditions. This variability was considered during the qualitative synthesis, with greater emphasis placed on evidence derived from well-replicated field experiments and studies with robust experimental designs.

## 3. Scope and Limitations of the Review

This review focuses on current knowledge and recent advances in INM strategies aimed at improving wheat productivity, grain quality, and micronutrient enrichment. The analysis emphasizes the roles of macronutrients and micronutrients, nutrient interactions, and agronomic biofortification approaches that contribute to sustainable wheat production systems. Attention is given to nutrient management practices influencing grain nutritional composition and nutrient-use efficiency under diverse agroecological conditions.

The scope of this review includes studies addressing the management of key macronutrients such as nitrogen (N), phosphorus (P), potassium (K), magnesium (Mg), and sulfur (S), as well as micronutrients including zinc (Zn) and selenium (Se), in addition to secondary macronutrients. The review also considers the influence of fertilization systems, genotype-specific responses, nutrient interactions, and abiotic stress conditions on wheat productivity and nutrient accumulation. In addition, this review examines the role of agronomic biofortification and modern breeding approaches in improving nutrient density in wheat grain. By integrating findings from different research areas, the review aims to provide a comprehensive understanding of how nutrient management strategies can enhance both yield and nutritional value in wheat-based production systems.

Despite these contributions, several limitations should be acknowledged. First, the review is based primarily on published peer-reviewed literature, which may exclude relevant information available in unpublished reports or grey literature. Second, variations in experimental conditions, soil characteristics, climatic factors, and wheat genotypes across studies may limit the direct comparability of results. Third, although the review discusses key nutrient interactions and management strategies, detailed molecular and physiological mechanisms underlying nutrient uptake and transport are addressed only briefly, as the primary focus remains on agronomic and production-oriented aspects of wheat nutrition.

Nevertheless, the synthesis presented in this review provides a broad overview of current research trends and highlights important areas for future investigation in INM and wheat nutritional improvement.

## 4. Integrated Nutrient Management and Nutritional Enhancement in Wheat

Integrated nutrient management (INM) in wheat has evolved from a conventional input-based approach into a more comprehensive strategy that simultaneously considers nutrient availability, genetic potential, and production environments to optimize both yield and grain nutritional quality. Increasing evidence indicates that crop performance is not determined by individual nutrient supply alone, but by the complex interactions among macro- and micronutrients, which regulate nutrient uptake efficiency, metabolic processes, and grain composition [24,25]. In parallel, agronomic biofortification strategies—particularly those involving zinc and selenium—have demonstrated substantial potential to enhance grain micronutrient density; however, their effectiveness remains strongly dependent on genotype-specific traits and environmental conditions [2,26].

**Figure 1:**
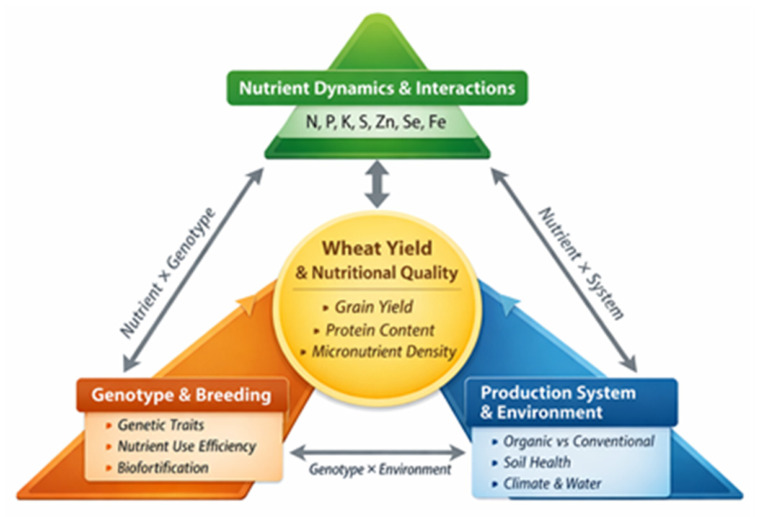
Triangular framework shows the interactions among nutrient dynamics, genotype, and production systems in determining wheat yield and grain nutritional quality.

Moreover, genotype × environment × management (G × E × M) interactions are increasingly recognized as key determinants of nutrient-use efficiency and crop nutritional outcomes across diverse agroecological conditions [27,28]. Integrated fertilization strategies combining mineral fertilizers, organic amendments, and biological inputs have been shown to improve nutrient synchrony, soil health, and overall system resilience [29,30]. Therefore, wheat nutritional enhancement can be more effectively understood through a coordinated perspective in which nutrient interactions, genetic factors, and production systems function as interdependent components rather than isolated drivers. This conceptual relationship is illustrated in Figure 1, which provides a unifying framework for understanding how these components collectively determine wheat yield and grain nutritional quality.

The conceptual framework presented in Figure 1 serves as the central structure guiding this review, linking nutrient interactions, genetic factors, and production systems across all subsequent sections.

### 4.1. Macronutrient Dynamics in Wheat

Within this framework, macronutrients represent the primary drivers of nutrient dynamics. Macronutrient management in wheat systems involves a complex network of interactions among nitrogen (N), sulfur (S), phosphorus (P), and potassium (K), which collectively regulate plant growth, nutrient assimilation, and grain quality. Rather than acting independently, these nutrients function through coordinated physiological and biochemical processes that determine nutrient-use efficiency and productivity. Nitrogen plays a central role in biomass accumulation and protein synthesis, while sulfur is essential for the formation of sulfur-containing amino acids and the optimization of nitrogen utilization efficiency [2,31].

The interaction between nitrogen and sulfur is particularly critical, as sulfur deficiency can limit nitrogen assimilation and reduce grain protein quality even under adequate nitrogen supply [2]. In addition, phosphorus contributes to energy transfer and root development, thereby enhancing nutrient acquisition, whereas potassium regulates osmotic balance, enzyme activation, and stress tolerance under variable environmental conditions [32,33]. These complementary and sometimes antagonistic interactions highlight the need for balanced nutrient management strategies that consider both individual nutrient roles and their interdependencies.

To facilitate a structured understanding of these relationships, macronutrient dynamics in wheat can be organized into key functional components, including nitrogen behavior, sulfur interactions, integrated N × S effects, and the combined roles of phosphorus and potassium, as illustrated in Figure S1 (Supplementary Materials). Within this conceptual framework, these macronutrients function as interconnected components that collectively regulate wheat growth and grain quality. Among them, nitrogen represents the central element due to its dominant role in biomass production, protein synthesis, and its strong interactions with other nutrients.

#### 4.1.1. Nitrogen

Nitrogen is the most yield-limiting nutrient in wheat production systems worldwide, playing a central role in chlorophyll synthesis, photosynthetic efficiency, enzyme activity, and overall biomass accumulation [34,35]. Adequate nitrogen supply enhances key yield components, including tiller formation, spike density, grain number per spike, and final grain yield. In addition, nitrogen availability directly influences grain protein concentration, making it a critical determinant of both productivity and end-use quality in wheat systems.

Despite its importance, NUE has shown limited improvement under intensive agricultural systems, largely due to excessive fertilizer inputs that exceed crop demand. This imbalance often results in diminished marginal yield responses and increased nitrogen losses through leaching, volatilization, and denitrification, posing significant environmental concerns [36]. Recent evidence highlights that improving NUE depends on synchronizing nitrogen supply with crop demand across key growth stages, particularly during stem elongation and grain filling [37]. From a physiological perspective, nitrogen uptake occurs primarily in the form of nitrate (NO_3_^−^) and ammonium (NH_4_^+^), which are assimilated via enzymatic pathways involving nitrate reductase and glutamine synthetase. The efficiency of these processes is closely linked to carbon metabolism and energy availability, emphasizing the integration of nitrogen assimilation within whole-plant metabolic networks [38]. The availability of nitrogen significantly interacts with other nutrients, particularly sulfur and zinc, thereby influencing both yield formation and grain nutritional quality [39,40]. The relationship between nitrogen and sulfur is especially critical, as sulfur is required for the synthesis of sulfur-containing amino acids that determine protein structure and functionality. In the absence of sufficient sulfur, nitrogen utilization efficiency declines, leading to the accumulation of non-protein nitrogen compounds rather than functional proteins. Moreover, nitrogen supply also affects micronutrient accumulation, including zinc, through its influence on root growth, transporter activity, and protein binding capacity. Recent studies indicate that optimized nitrogen management can enhance micronutrient accumulation in wheat grains, although excessive nitrogen may dilute mineral concentrations due to yield-driven effects [41].

Overall, nitrogen plays a pivotal yet highly interactive role in wheat production systems, where its efficiency and impact are strongly regulated by physiological processes, nutrient interactions, and management practices. This highlights the need for integrated nutrient strategies that improve NUE while maintaining grain quality and environmental sustainability.

Given the close metabolic interdependence between nitrogen and sulfur, understanding sulfur dynamics is essential to fully interpret NUE and its effects on grain quality.

#### 4.1.2. Sulfur and the Critical Nitrogen–Sulfur (N × S) Interaction

Sulfur plays a fundamental role in plant metabolism through its involvement in the synthesis of sulfur-containing amino acids (cysteine and methionine), vitamins, and essential coenzymes, thereby contributing to the incorporation of nitrogen into functional proteins [35]. In wheat systems, sulfur availability is closely linked to nitrogen metabolism, as it regulates the formation of structurally and functionally important proteins required for grain development and end-use quality. Under sulfur-deficient conditions, absorbed nitrogen tends to accumulate in non-protein forms such as nitrate and soluble amines, resulting in reduced nitrogen-use efficiency (NUE) and inferior grain quality [39].

The relationship between nitrogen and sulfur can be described as a co-regulated metabolic interaction, as both nutrients are functionally interconnected through shared biochemical pathways. Nitrogen assimilation depends on adequate sulfur supply to support the synthesis of sulfur-rich proteins and enzymes, while sulfur assimilation requires sufficient nitrogen to sustain overall metabolic activity. This coordination is mediated by key biochemical processes, including sulfate reduction, cysteine biosynthesis, and glutathione metabolism [42,43].

A balanced supply of N and S has been shown to enhance protein synthesis, improve nitrogen remobilization during grain filling, and increase yield stability across diverse agroecological conditions [5]. Meta-analytical and field-based evidence (Table 1 and Table S1) indicates that combined nitrogen and sulfur application is associated with yield increases of approximately 4–18% and grain protein increases of 2–6%, depending on environmental conditions and sulfur availability [23,39]. Lower response magnitudes are generally reported in large-scale multi-environment analyses, whereas higher responses are observed under sulfur-deficient conditions or optimized fertilization strategies. Variability in reported responses reflects differences in soil sulfur status, nitrogen management, environmental conditions, and genotype, as detailed in Supplementary Table S1 (Supplementary Materials).

These responses are particularly pronounced under low soil sulfur availability (e.g., <7.2 mg kg^−1^), in conservation or no-tillage systems, and in crop rotations involving legumes, where nutrient mineralization dynamics strongly influence sulfur availability.

Field and meta-analytical studies consistently indicate that combined nitrogen and sulfur fertilization provides more stable and consistent improvements than nitrogen-only management, particularly in calcareous and sandy soils where sulfate availability is limited [5,39]. In such environments, sulfur supplementation improves nitrogen assimilation efficiency, enhances root nutrient uptake, and mitigates imbalances associated with sulfur limitation [44,45]. In contrast, nitrogen-only responses are more variable and highly environment-dependent [46,47].

In addition to yield effects, sulfur plays a critical role in determining wheat processing quality by regulating the balance between gliadins and glutenin. Adequate sulfur supply promotes glutenin synthesis, thereby improving dough strength and baking performance, whereas sulfur deficiency leads to protein imbalances that negatively affect functional quality. Recent evidence further highlights the importance of optimizing the N × S ratio to achieve both yield and grain quality objectives [48].

Response ranges are derived directly from reported values across selected meta-analyses and field studies and are presented as indicative ranges rather than statistically aggregated estimates. Detailed supporting data, including individual study values and variability, are provided in Supplementary Table S1 (Supplementary Materials).

Overall, the N × S interaction represents a central component of macronutrient management in wheat, where coordinated nutrient supply contributes to improved nitrogen efficiency, enhanced grain nutritional quality, and greater system stability under variable environmental conditions.

#### 4.1.3. Nitrogen–Sulfur Interaction and Wheat Grain Quality

The concentration of protein in wheat grains is mainly influenced by nitrogen availability, while the composition and functionality of the protein are significantly affected by sulfur supply [1]. A lack of sulfur disrupts the equilibrium between gliadin and glutenin fractions, leading to a decreased production of S-rich glutenin in comparison to S-poor gliadins. This results in diminished gluten networks and subpar dough characteristics even with high overall protein content [5].

Combined nitrogen and sulfur fertilization is associated with improved gluten strength, dough stability, and bread-making quality through enhanced synthesis of high-molecular-weight glutenin subunits and promotion of disulfide bond formation. Evidence summarized in Table 1 and Table S1 (Supplementary Materials) indicates that increases in grain protein concentration under combined fertilization typically range between 2% and 6%, depending on sulfur availability and environmental conditions [5,39,43]. The ideal N:S ratio for quality in bread-making is roughly between 7:1 and 10:1, varying with genotype and environmental factors.

#### 4.1.4. Phosphorus (P) and Potassium (K)

Phosphorus plays a vital role in energy metabolism (ATP production), photosynthesis, the synthesis of nucleic acids, and root growth. It aids in spikelet growth and grain filling by preserving membrane integrity and facilitating energy transfer processes [35]. P deficiency restricts biomass growth, decreases tiller survival rates, and might negatively affect protein and starch content.

Potassium controls osmotic equilibrium, stomatal permeability, enzyme activation, and resilience to stress [49]. Sufficient K enhances water-use efficiency, grain development, and protein quality by boosting N movement and assimilation. K deficiency is especially harmful during heat or drought stress, lowering both yield and nutrient levels. Recent findings emphasize K × Si synergy, in which silicon boosts the expression of K^+^ transporter TaAKT1 under heat stress, facilitating osmotic stability and photosynthesis [50].

While macronutrients form the fundamental basis of wheat growth and productivity, their role extends beyond yield formation to influencing the uptake, translocation, and accumulation of micronutrients in the grain. This interdependence highlights that optimizing macronutrient management is a prerequisite for effective micronutrient enrichment. Within the conceptual framework presented in Figure 1, this transition reflects the shift from primary nutrient-driven processes to targeted strategies for improving grain nutritional quality through micronutrient biofortification.

### 4.2. Micronutrient Biofortification: Agronomic and Genetic Approaches

Micronutrient biofortification represents a key component of the framework, linking nutrient management with nutritional outcomes. Micronutrient malnutrition (Zn, Se, Fe), along with inadequate magnesium (Mg) intake as a macronutrient, affects billions globally, particularly in populations dependent on cereal-based diets. Recent global assessments further emphasize that biofortification responses must be interpreted within a broader agroecological framework, as nutrient accumulation in wheat is strongly influenced by genotype × environment × management interactions [51]. This highlights the need for integrated agronomic and genetic approaches while also recognizing variability in nutrient bioavailability and regional implementation.

#### 4.2.1. Zinc (Zn) Biofortification

Zinc (Zn) deficiency is widely prevalent in populations relying on wheat-based diets, particularly in regions dominated by calcareous and alkaline soils where Zn availability is inherently low [2]. Enhancing Zn concentration in wheat grain therefore requires an integrated approach that considers soil properties, nutrient management, and genotypic variation.

The concentration of Zn in wheat grain is strongly influenced by soil Zn availability, plant genotype, and nutrient interactions. Recent global evidence emphasizes that Zn biofortification outcomes vary significantly across agroecological zones and management systems, reinforcing the importance of context-specific interpretation [51]. Among these, nitrogen (N) fertilization plays a key synergistic role. Nitrogen improves root system development and stimulates the production of Zn-chelating compounds such as phytosiderophores, which enhance Zn solubility, uptake, and translocation to the grain [40]. This interaction highlights the importance of coordinated nutrient management strategies rather than single-nutrient interventions.

In addition to soil application, foliar fertilization has proven to be an effective strategy for Zn enrichment [2,13]. A two-year field study on durum wheat demonstrated that the combined foliar application of Zn and selenium (Se) significantly increased grain Zn concentration by 1.44-fold and Se concentration by 3.41-fold compared to the control. This treatment also resulted in a 15% increase in grain yield, indicating a strong synergistic effect between Zn and Se on both nutritional quality and productivity [2,13].

**Figure 2:**
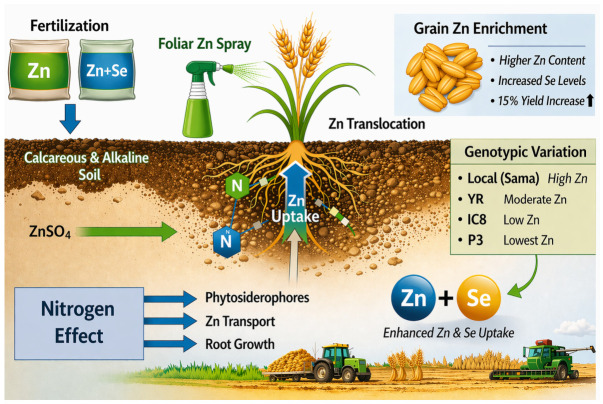
Schematic diagram of the mechanisms and interacting factors controlling zinc (Zn) bio-fortification in wheat, including soil availability, nitrogen interaction, fertilization practices, genotypic variation, and Zn–Se synergistic effects on grain Zn accumulation and yield. Some graphical elements were generated using AI-assisted tools and subsequently modified and integrated by the authors.

Fertilization practices further modulate Zn accumulation. Organic fertilization resulted in significantly higher grain Zn concentrations compared to conventional fertilization across all genotypes [52]. This enhancement is likely attributed to increased soil biological activity and improved micronutrient availability associated with organic amendments, including their role in lowering soil pH and enhancing Zn solubility [40,41]. The interaction between genotype and fertilization system is illustrated in Figure 2, which conceptually summarizes the pathways and factors influencing Zn uptake, translocation, and accumulation in wheat grain.

To clarify the conceptual representation in Figure 2, Zn uptake refers specifically to the absorption of Zn^2+^ ions by root systems from the rhizosphere, followed by their transport to aerial parts (shoots) via the xylem. The term “Zn uptake in shoots” therefore reflects the net accumulation of Zn in aboveground tissues after root absorption and internal translocation. Although selenium (Se) pathways are not explicitly illustrated as independent processes, the figure highlights the synergistic interaction between Zn and Se through the combined arrow, indicating their co-enhanced uptake under integrated fertilization strategies. This interaction is supported by evidence that Zn nutrition can improve root activity and transporter expression, indirectly facilitating Se assimilation.

Phytosiderophores are intentionally indicated as a key mechanism under nitrogen (N) influence, as N availability enhances their synthesis and release, thereby improving Zn solubility and transport in calcareous soils. The repeated mention emphasizes their dual role in both mobilization and transport processes.

The green arrows represent nutrient flow direction and biological facilitation processes, particularly those enhanced by fertilization inputs and soil–plant interactions.

The placement of foliar Zn spray in the soil compartment is schematic rather than literal, intended to indicate its contribution to overall plant Zn status alongside soil-applied Zn (ZnSO_4_), rather than its physical application pathway.

#### 4.2.2. Selenium (Se) Biofortification

Selenium (Se) is an essential micronutrient for human health, playing a critical role in antioxidant defense systems and immune function [53,54]. However, the concentration of Se in wheat grain is largely dependent on soil Se availability, which varies significantly across geographic regions, leading to pronounced disparities in dietary Se intake [55].

In agricultural systems, selenium is predominantly present in the form of selenate (SeO_4_^2−^), which is highly soluble and readily available for plant uptake. Agronomic biofortification through the application of Se fertilizers, particularly as selenate, represents an effective and direct strategy to enhance Se concentration in wheat grain [54,56]. Once absorbed by roots, Se is efficiently translocated via the xylem to aerial plant parts and subsequently redistributed to developing grains, making it suitable for grain biofortification. The efficiency of Se uptake and accumulation is strongly influenced by soil properties, fertilization practices, and plant genotype. Soil organic matter and microbial activity play a crucial role in regulating Se availability through processes such as mineralization and transformation between different Se species. Comparative studies between organic and conventional farming systems have demonstrated that organic fertilization can significantly increase grain Se concentration, even when associated with relatively lower grain yields. This effect is primarily attributed to enhanced biological activity and improved micronutrient availability under organic management systems [52].

The interaction between genotype and fertilization system played a decisive role in determining Se accumulation patterns. In most genotypes, organic fertilization significantly enhanced Se concentration compared to conventional fertilization. However, Sids12 showed a contrasting response, with higher Se concentration under conventional fertilization, highlighting genotype-specific differences in Se uptake efficiency and internal utilization. This behavior underscores the importance of selecting appropriate genotypes tailored to specific agronomic conditions. Recent evidence further indicates that the biological activity and nutritional functionality of Se in plants is strongly dependent on its chemical form (speciation), with organic Se compounds such as selenomethionine (SeMet), selenocystine (SeCys_2_), and Se-methylselenocysteine (MSC) exhibiting higher bioactivity and lower toxicity compared to inorganic forms (selenate and selenite). Plants serve as major sources of these organic Se species, converting and storing absorbed Se into bioavailable organic forms, thereby enhancing their nutritional value for humans [57].

The combined effects of soil Se forms, fertilization strategies, and genotypic variation on Se uptake, translocation, and grain enrichment are conceptually illustrated in Figure S2 (Supplementary Materials), which summarizes the key pathways regulating selenium biofortification in wheat.

#### 4.2.3. Magnesium (Mg) Nutrition

**Figure 3:**
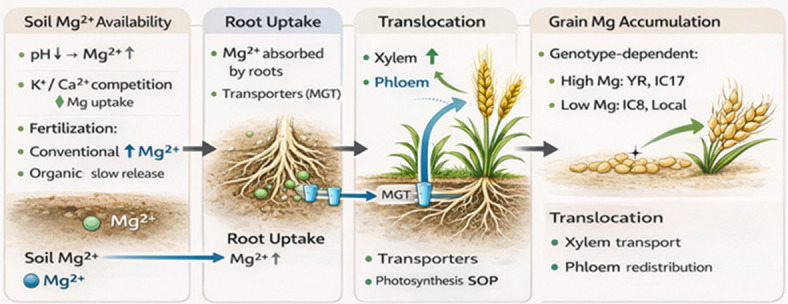
Schematic diagram of magnesium (Mg^2+^) dynamics in wheat, illustrating soil Mg^2+^ availability, root uptake via Mg transporters (MGT), translocation through xylem and phloem, and genotype-dependent grain accumulation. Soil factors (pH, K^+^/Ca^2+^ competition, and fertilization systems) regulate Mg^2+^ availability, uptake efficiency, and internal redistribution within the plant. Some graphical elements were generated using AI-assisted tools and subsequently modified and integrated by the authors.

Magnesium availability in soil is governed by a complex interplay of physicochemical and agronomic factors, including soil pH, cation competition, and fertilization practices. Soil pH plays a particularly critical role in regulating Mg solubility and mobility, where moderate acidification generally enhances Mg availability by increasing its dissolution from soil minerals. In this context, nitrogen fertilization—especially in the form of urea—can indirectly improve Mg availability through soil acidification processes that promote Mg release into the soil solution [58,59,60,61,62,63]. However, this effect is not universally beneficial, as excessive acidification may also increase leaching losses in coarse-textured soils, thereby reducing Mg retention and long-term availability. These soil-related processes represent the initial stage controlling Mg^2+^ availability in the soil system, as illustrated in Figure 3.

In addition to pH effects, Mg dynamics in soil are strongly influenced by competitive interactions with other cations, particularly potassium (K^+^) and calcium (Ca^2+^). High levels of K fertilization, commonly applied in intensive wheat production systems, may suppress Mg uptake due to competitive inhibition at root uptake sites. Similarly, elevated Ca concentrations can reduce Mg availability through cation exchange processes in the soil matrix. These antagonistic interactions highlight that Mg nutrition cannot be considered in isolation but must be evaluated within the broader framework of INM, where nutrient balance rather than absolute supply determines plant uptake efficiency. Such interactions directly influence the transition from soil Mg availability to root uptake efficiency (Figure 3). Fertilization systems further modulate Mg availability through differences in nutrient release patterns and soil biochemical processes. Conventional fertilization systems often provide more readily available Mg due to the rapid dissolution of mineral fertilizers, leading to immediate increases in soil solution Mg concentrations. In contrast, organic fertilization systems rely on mineralization and organic matter decomposition, resulting in a slower and more gradual release of Mg [64,65,66]. While this slower release may limit short-term Mg availability, it can contribute to improved soil structure, enhanced cation exchange capacity, and greater long-term nutrient buffering. Therefore, the apparent superiority of conventional systems in increasing Mg availability may be context-dependent and influenced by soil type, organic matter content, and management intensity. These contrasting fertilization effects are schematically represented in the soil compartment of Figure 3. Agronomic management practices strongly influence Mg uptake and accumulation in wheat, as demonstrated by Abd-Elmoniem et al. [67], who evaluated seven wheat genotypes under conventional and organic fertilization systems. Their findings indicated significantly higher Mg concentrations under conventional fertilization, which can be attributed to increased nutrient availability and enhanced root uptake efficiency under conditions of higher Mg solubility. However, it is important to note that such responses may vary across environments, particularly under conditions were nutrient imbalances or soil constraints limit Mg mobility or root accessibility. Consequently, the observed advantages of conventional fertilization should be interpreted within specific soil and management contexts rather than generalized across all production systems. This stage corresponds to the root uptake processes mediated by Mg transporters.

At the physiological level, Mg plays a central role in phloem loading and assimilating transport, directly influencing carbohydrate partitioning from source leaves to developing grains. Adequate Mg supply facilitates efficient translocation of photosynthates, thereby supporting grain filling and yield formation. Conversely, Mg deficiency disrupts photosynthetic activity, impairs phloem transport, and leads to the accumulation of carbohydrates in source tissues, ultimately reducing assimilate availability for grain development. These physiological constraints further emphasize the importance of maintaining balanced Mg nutrition within INM systems. As highlighted by Gerendás and Führs [68], the largest improvements in crop quality are observed when Mg nutrition is corrected from deficient to adequate levels, because Mg deficiency severely restricts photosynthesis, phloem loading, and assimilate translocation to the grain. Once Mg supply is improved, these constraints are rapidly alleviated, leading to disproportionally large gains in yield formation and quality-related traits compared with situations where Mg is already sufficient [69]. In addition to absolute Mg supply, competitive interactions with other cations—especially high Ca^2+^ and K^+^ levels—can considerably suppress Mg^2+^ uptake at root transport sites and via cation exchange in the rhizosphere, leading to Mg deficiency symptoms despite seemingly adequate soil Mg status [68,70].

Nitrogen management also has implications for process-related contaminants. Elevated N fertilization, especially when not balanced with adequate sulfur, is associated with increased concentrations of free asparagine in wheat grain [71,72]. Because free asparagine is the main amino acid precursor for acrylamide during high-temperature baking, excessive N inputs may indirectly increase acrylamide formation in wheat-based products. This underscores the need for balanced N and S supply to optimize both nutritional quality and food safety outcomes.

The integrated effects of soil properties, fertilization practices, nutrient interactions, and genotypic variation on Mg uptake, translocation, and accumulation in wheat grain are conceptually illustrated in Figure 3. The figure highlights key processes including soil Mg availability, the influence of soil pH and cation competition, root uptake mechanisms, physiological functions in photosynthesis and metabolism, and genotype-dependent efficiency under contrasting fertilization systems. These processes are clearly organized into four sequential components: soil Mg availability, root uptake, translocation, and grain accumulation, providing a simplified and structured overview of Mg dynamics in wheat.

While micronutrient biofortification strategies at the element level provide effective means to enhance grain nutritional quality, their overall efficiency is strongly influenced by the broader production system in which they are implemented. Differences in nutrient availability, soil biological activity, and management intensity across production systems can significantly modify micronutrient uptake, translocation, and accumulation. Therefore, a system-level perspective is essential to fully understand how biofortification strategies perform under contrasting agricultural conditions.

### 4.3. System-Specific Responses: Organic Versus Conventional Production

Wheat production systems managed under organic and conventional regimes exhibit distinct nutrient cycling processes, soil biological interactions, and overall crop performance due to differences in nutrient availability, fertilizer inputs, and management intensity. Organic systems typically rely on organic amendments and biologically mediated nutrient release, which can enhance soil structure and microbial activity but often results in slower nutrient mineralization and reduced synchronous supply of nitrogen and other key macro- and micronutrients during critical growth stages [73,74]. In contrast, conventional systems provide readily available mineral nutrients that align more closely with plant demand for rapid vegetative growth and grain filling, frequently resulting in higher yields but sometimes at the expense of soil biological complexity [75,76]. Meta-analyses comparing organic and conventional systems across multiple crops, including cereals such as wheat, have reported a consistent yield gap favoring conventional management (on average up to 25–30% lower yields in organic systems), reflecting the challenge of synchronizing nutrient release with crop demand in organic production. However, some recent field trials also highlight that with optimized organic amendments and integrated approaches, organic systems can maintain yields and improve certain quality traits, such as protein and micronutrient concentrations, while supporting soil health and ecosystem sustainability [75,77]. This complex interplay between nutrient supply dynamics, crop response, and ecosystem services underscores the need to examine system-specific responses when comparing yield performance and nutritional outcomes in wheat across contrasting production systems.

#### 4.3.1. Comparative Performance

Wheat production systems exhibit fundamentally different nutrient dynamics that translate into contrasting outcomes in grain yield and nutritional quality. Across diverse environments, conventional fertilization systems consistently outperform organic systems in terms of yield, largely due to the immediate availability of mineral nutrients particularly nitrogen which directly supports rapid canopy development, photosynthetic capacity, and grain filling. Empirical evidence from Saudi Arabian agroecosystems confirms this pattern, where conventional systems produced substantially higher grain yields compared to organic management across multiple genotypes and growing seasons [30,52,67].

This yield advantage is primarily driven by differences in nutrient release kinetics and synchronization with crop demand. Mineral fertilizers provide readily available nutrients that coincide with critical growth stages, whereas organic amendments depend on microbial mineralization processes that are inherently slower and influenced by environmental conditions [78,79,80]. Recent studies further confirm that nitrogen limitation during early growth stages remains a key constraint in organic systems, leading to reduced tiring and lower yield potential [81,82,83].

Despite these yield limitations, organic systems consistently enhance grain micronutrient concentrations, particularly zinc and selenium, as demonstrated by significantly higher concentrations under organic fertilization compared to conventional systems [52]. This enrichment is attributed to improved soil biological activity, enhanced rhizosphere processes, and increased availability of micronutrients through organic complexation and gradual nutrient release. Organic matter decomposition produces low-molecular-weight organic acids that reduce soil pH locally and increase micronutrient solubility, while microbial activity facilitates nutrient cycling and mobilization [59,84].

Recent research provides strong support for these mechanisms, highlighting that organic systems promote greater microbial diversity, enzyme activity, and nutrient turnover, all of which contribute to improved micronutrient availability [85,86,87]. In addition, organic ligands play a critical role in chelating micronutrients such as Zn and Fe, enhancing their bioavailability for plant uptake [88,89]. Lower reliance on highly soluble mineral fertilizers further reduces competitive ion interactions in the soil solution, thereby facilitating micronutrient uptake [90]. Collectively, these findings indicate that the contrast between organic and conventional systems reflects a fundamental trade-off between nutrient quantity and nutrient quality, where rapid nutrient supply in conventional systems favors yield, while biologically mediated nutrient cycling in organic systems enhances nutritional density. However, this dichotomy is not absolute and can be modulated through improved management strategies and genotype selection.

#### 4.3.2. Genotype × System Interactions

The differential performance of wheat genotypes across production systems highlights the importance of genotype × environment × management interactions in determining both yield stability and nutritional outcomes. Long-term multi-environment trials demonstrate that genotypes vary widely in their ability to adapt to contrasting nutrient regimes, with some exhibiting broad adaptability while others show strong system-specific responses [91].

Analytical approaches such as AMMI and GGE biplot models provide valuable insights into these interactions by partitioning variation into genotype effects, environmental influences, and their interactions. Results indicate that environmental factors, including fertilization system and seasonal variability, account for a substantial proportion of yield variation, while genotype-specific responses determine stability and adaptability across conditions. The significant contribution of interactive principal component axes (IPCA) underscores the complexity of these relationships and the necessity of multi-dimensional evaluation frameworks.

In this context, stability analysis reveals that certain genotypes, such as IC8, combine high yield potential with low variability across environments, indicating strong adaptability to both organic and conventional systems. In contrast, other genotypes exhibit higher instability, suggesting specific adaptation to nutrient regimes. These findings are consistent with broader studies demonstrating that genotype performance under low-input or organic conditions is strongly influenced by traits such as root architecture, nutrient uptake efficiency, and symbiotic interactions with soil microbiota [92,93].

The GGE biplot analysis further illustrates the presence of distinct mega-environments corresponding to organic and conventional systems, each favoring different genotypes. As conceptually illustrated in Figure S3 (Supplementary Materials), genotypes such as P5 and IC17 exhibit superior performance under conventional fertilization, whereas IC8 demonstrates clear dominance under organic conditions, reflecting its enhanced efficiency in nutrient acquisition and utilization under limited nutrient availability [91]. This separation of mega-environments emphasizes that genotype selection cannot be generalized across systems but must be tailored to specific management conditions.

Importantly, the integration of yield potential with stability metrics provides a more robust framework for cultivar selection than reliance on yield alone. The use of combined selection criteria, incorporating both mean performance and stability indices, has been widely recommended for breeding programs targeting diverse and variable environments [94]. Recent advances further suggest that incorporating physiological and genomic traits into selection models can improve prediction accuracy and enhance breeding efficiency [95,96,97].

Overall, genotype × system interactions represent a critical dimension of wheat production, where the alignment between genetic traits and management practices determines system performance. Optimizing this alignment through targeted breeding and system-specific management strategies offers significant potential to reduce the yield gap between organic and conventional systems while maintaining or enhancing grain nutritional quality.

While system-specific responses highlight how different production environments influence wheat yield and nutritional outcomes, a deeper understanding of these variations requires examining the underlying nutrient interaction mechanisms. The complexity of genotype × system responses is largely governed by synergistic and antagonistic relationships among nutrients, which regulate their availability, uptake, and utilization. Therefore, integrating these interactions into a unified analytical framework provides critical insights into the processes driving system-level performance and nutritional quality.

While the system-specific framework presented here integrates evidence from multiple studies included in this review, a substantial portion of the underlying data originates from arid and semi-arid agroecosystems, particularly those characterized by calcareous soils. Under such conditions, nutrient dynamics are strongly influenced by high soil pH, carbonate content, and limited moisture availability, which directly affect nutrient solubility, precipitation reactions, and plant uptake efficiency. Consequently, the strength and direction of certain interactions (e.g., P–Zn, Ca–P, and Fe availability constraints) may differ under contrasting soil and climatic conditions. In more humid or temperate regions, including the North China Plain, European cropping systems, and North American production environments, higher organic matter content, different pH regimes, and distinct moisture dynamics can alter nutrient cycling pathways and interaction patterns. Therefore, while the conceptual basis of nutrient interactions and system-specific responses remains broadly applicable, quantitative responses and management implications should be interpreted within the context of local soil and environmental conditions and may require region-specific calibration.

### 4.4. Nutrient Interaction Matrix: Synergies and Antagonisms

Recent advances in plant nutrition research have moved beyond single-nutrient perspectives toward integrated conceptual frameworks that capture the complexity of nutrient interactions within cropping systems. In wheat, these interactions are synthesized into a conceptual nutrient interaction matrix (Figure S4), representing hypothesized synergistic and antagonistic relationships among key macro- and micronutrients based on evidence reported in the literature. This matrix provides a schematic visualization of nutrient interaction patterns, emphasizing that crop performance is governed not only by nutrient availability but also by the nature of inter-nutrient relationships. In this framework, only pairwise interactions between different nutrients are considered, while diagonal elements (self-interactions) are excluded from interpretation.

As illustrated in Figure S4, nutrient interactions are distributed along a continuum from strong synergistic effects to pronounced antagonisms. The most prominent synergistic interaction is observed between nitrogen and sulfur (N × S), reflecting their tightly coordinated roles in amino acid synthesis and protein assembly. Sulfur availability directly regulates the formation of sulfur-containing amino acids and disulfide bonds in gluten proteins, thereby enhancing nitrogen assimilation efficiency and its remobilization to developing grains [98]. This relationship is widely reported in the literature as a strong synergistic interaction, indicating that improved sulfur nutrition enhances nitrogen assimilation and its utilization in protein synthesis and grain development. Similar conclusions have been reported in recent physiological and proteomic studies demonstrating that nitrogen–sulfur co-regulation governs grain protein composition and functionality [2,33].

In addition to N × S, strong synergistic interactions are evident between potassium and silicon (K × Si) and between potassium and calcium (K × Ca). The K × Si interaction has been associated with enhanced activation of potassium transport systems, including TaAKT1, under heat stress conditions, resulting in improved osmotic regulation and increased grain set [52]. Silicon acts as a functional modulator of nutrient transport and stress signaling pathways, thereby enhancing potassium use efficiency and overall plant resilience [99,100]. Similarly, the interaction between potassium and calcium contributes to membrane stability, ion homeostasis, and stomatal regulation, reinforcing plant tolerance to transient environmental stresses.

Moderate synergistic interactions further highlight the integrative nature of nutrient dynamics in wheat systems. The nitrogen–zinc interaction (N × Zn) enhances micronutrient mobility within the plant, as nitrogen supply increases the synthesis of organic ligands that facilitate zinc transport through the phloem, resulting in improved grain enrichment [36]. Likewise, the phosphorus–sulfur interaction (P × S) reflects coordinated regulation of nutrient uptake mechanisms, including increased root acid-phosphatase activity and co-expression of phosphate and sulfate transporters, which together improve nutrient acquisition efficiency. These findings align with recent studies emphasizing the importance of coordinated nutrient signaling networks in optimizing plant nutrition [39,101].

Conversely, the interaction matrix also highlights critical antagonisms that can constrain nutrient availability and uptake. The strongest antagonistic interaction is observed between calcium and phosphorus (Ca × P), where high phosphorus availability promotes the formation of insoluble calcium–phosphate complexes in the rhizosphere, thereby reducing the availability of both nutrients [98]. This mechanism is particularly relevant in calcareous soils, where precipitation reactions are rapid and can significantly limit nutrient efficiency. Moderate antagonistic interactions, including phosphorus–zinc (P × Zn) and calcium–zinc (Ca × Zn), further demonstrate the competitive dynamics that arise under imbalanced fertilization regimes. These interactions are well-documented in soil chemistry and plant nutrition literature, where excessive phosphorus supply is known to inhibit zinc uptake through both chemical and physiological pathways [102].

Notably, the interaction matrix does not indicate strong or moderate pairwise interactions involving iron (Fe) under the represented conditions, suggesting that Fe dynamics may be more strongly influenced by soil redox conditions and plant-specific uptake strategies than by direct nutrient competition within this interaction framework. This observation is consistent with findings that iron availability is primarily regulated by rhizosphere processes such as acidification and chelation rather than by simple nutrient interactions [103].

From an agronomic perspective, integrating these interaction patterns into nutrient management strategies offers significant potential for improving both productivity and sustainability. Balanced multi-nutrient approaches informed by interaction matrices can enhance NUE and reduce unnecessary fertilizer inputs. Life cycle assessment studies indicate that optimizing nutrient interactions can reduce total input requirements by 10–15% without compromising yield, while simultaneously lowering the carbon footprint of wheat production systems [104]. Furthermore, leveraging beneficial interactions such as K × Si provides an alternative pathway for enhancing stress tolerance without increasing nitrogen inputs, which is particularly relevant under climate change scenarios characterized by rising temperatures and increased environmental variability.

Overall, the nutrient interaction matrix presented in Figure S4 (Supplementary Materials) provides a conceptual framework for understanding nutrient interaction patterns in wheat systems. By shifting the focus from isolated nutrients to interaction-based interpretations, this approach supports more integrated thinking in nutrient management strategies aimed at improving productivity and grain quality.

### 4.5. Breeding Acceleration and Genotype × Management Integration

Breeding strategies represent the genetic dimension of the proposed framework. The acceleration of wheat breeding has become a central focus in modern crop improvement programs due to the urgent need to enhance yield, grain nutrition, and stress resilience within shortened timeframes. Traditional breeding cycles often span a decade or more, limiting the responsiveness of breeding pipelines to emerging challenges such as climate change, evolving pathogen pressures, and nutritional demands. To address these constraints, breeding methodologies have evolved substantially over recent decades, integrating molecular, genomic, and biotechnological innovations that collectively reduce generational turnover and improve selection accuracy [105]. Combined approaches that integrate generation acceleration systems such as speed breeding with high-throughput phenotyping, genomic selection, and marker-assisted techniques are demonstrating remarkable efficiency gains in wheat, enabling breeders to fast-track generational advancement while retaining rigorous selection standards [106]. Alongside these systems, genomic tools such as dense SNP arrays, genome-wide association studies (GWAS), and multi-omics platforms have expanded the range of traits that can be dissected and selected at early stages of breeding programs, enhancing both yield potential and nutrient-use efficiency [107]. At the same time, advances in precise genome editing technologies, particularly CRISPR/Cas systems, are ushering in a new era where targeted modification of key genes associated with nutrient partitioning, stress tolerance, and metabolic efficiency can be achieved with high precision and reduced linkage drag [108]. Collectively, these innovations illustrate a shift from phenotype-driven approaches to integrated, data-driven breeding frameworks that significantly compress breeding timelines and expand the genetic toolkit available for nutrient enhancement and quality traits in wheat.

#### 4.5.1. Technological Evolution in Wheat Breeding

Recent evidence indicates that the average release time for nutrient-enhanced wheat cultivars has decreased substantially, from approximately 14 years during the marker-assisted selection (MAS) era to less than 5 years in the post-2019 period [109]. This acceleration coincides with the expansion of CRISPR-based pipelines in several countries, including Argentina and Australia, and reflects a fundamental shift from indirect selection approaches, such as QTL tagging, to direct allele engineering of genes controlling nutrient partitioning.

**Figure 4:**
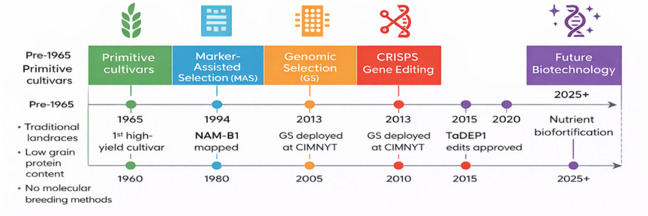
Evolution of wheat breeding technologies from traditional cultivars to modern biotechnology approaches for nutrient biofortification.

Figure 4 illustrates the chronological development of wheat breeding strategies from traditional landrace cultivation to advanced molecular biotechnology approaches. Prior to 1965, wheat improvement relied mainly on primitive cultivars and traditional landraces, which were characterized by relatively low grain protein content and the absence of molecular breeding techniques [110]. The introduction of the first high-yielding cultivars around 1965 marked a major turning point in wheat improvement.

Subsequent advances in molecular genetics led to the emergence of marker-assisted selection (MAS) during the 1980s and 1990s, highlighted by the mapping of the NAM-B1 gene in 1994, which is associated with enhanced nutrient remobilization and increased grain protein content. The development of genomic selection (GS) further accelerated breeding efficiency, particularly with its large-scale deployment at international research centers such as CIMMYT around 2013, enabling genome-wide prediction of breeding values.

More recently, genome editing technologies, particularly CRISPR-based approaches, have enabled precise and targeted modification of key genes associated with yield, nutrient-use efficiency, and grain quality. For instance, gene edits involving TaDEP1 demonstrated the potential to improve yield-related traits and plant architecture without compromising productivity [18].

The integration of advanced biotechnological tools is expected to further enhance nutrient biofortification and precision breeding strategies beyond 2025. These developments aim to improve micronutrient density, grain quality, and overall productivity in wheat under changing environmental conditions. Overall, this progression reflects a clear transition from phenotype-based selection to integrated, data-driven genomic and biotechnological breeding approaches.

#### 4.5.2. Current Frontiers: Gene Editing for Nutrient Efficiency

Recent advances in genome editing technologies, particularly CRISPR/Cas systems, have revolutionized wheat breeding by enabling precise modification of genes controlling nutrient uptake, assimilation, and partitioning. Unlikemilarly, the interaction between potassium and calcium contributes to membrane stability, io conventional breeding or marker-assisted selection, genome editing allows direct manipulation of alleles associated with NUE, grain micronutrient accumulation, and quality traits, significantly shortening breeding cycles while maintaining or improving yield potential [111]. This shift represents a critical frontier in integrating genetic potential with tailored management practices to maximize both productivity and grain nutritional quality.

Several experimental and molecular studies illustrate this potential. Reduction in RNA levels of multiple NAM homologs through RNA interference has been shown to delay senescence by more than three weeks and reduce wheat grain protein, zinc, and iron concentrations by over 30%, highlighting the central role of NAC transcription factors in nutrient remobilization and grain nutritional quality [112]. In parallel, TaDEP1 has been identified as an important regulator of wheat growth and development, and advances in CRISPR/Cas9 genome editing enable efficient and transgene-free modification of such target genes, providing new opportunities to optimize nutrient allocation and improve agronomic performance in wheat [113]. Similarly, TaVIT2 overexpression lines demonstrate increased bioavailable iron concentrations in white flour, confirming that targeted gene modifications can directly influence micronutrient density [18,111,112,113,114]. Field evaluations further indicate that NUE gains from edited lines plateau without concurrent adjustments in fertilizer timing, formulation, and soil management, emphasizing the need for holistic G × E × M frameworks that combine genetic editing with agronomic optimization [64,107].

Beyond these specific examples, genome editing also enables simultaneous targeting of multiple loci involved in nutrient transport, metabolism, and storage, expanding the range of traits accessible for improvement. Integration of CRISPR/Cas technologies with speed breeding, high-throughput phenotyping, and genomic selection has accelerated trait fixation, allowing breeders to compress timelines from over a decade to under five years for nutrient-enhanced cultivars [106]. Moreover, ongoing research is exploring the deployment of genome editing in multi-location trials to validate nutrient efficiency gains across diverse environments, ensuring the stability of trait expression under variable conditions. Collectively, these developments underscore that gene editing is a transformative tool for nutrient efficiency in wheat, not only enhancing grain protein and micronutrient content but also aligning with INM strategies. When combined with optimized fertilization, root architecture improvements, and environment-specific management, edited wheat lines can achieve their full genetic potential while contributing to sustainable and resilient crop production systems.

While advances in breeding and genome editing provide powerful tools to enhance nutrient efficiency and grain quality, their full potential can only be realized when integrated within a broader system-level perspective. This highlights the need for a comprehensive synthesis that links genetic innovation with agronomic management and environmental factors to address the yield–quality trade-offs in wheat production.

### 4.6. Synthesis and Future Perspectives

#### 4.6.1. Yield–Quality Trade-Offs

Field studies conducted under Saudi Arabian agroecological conditions [52,67,91] consistently demonstrate a fundamental trade-off between grain yield and micronutrient density in wheat production systems. As illustrated in Figure S5 (Supplementary Materials), conventional fertilization regimes are generally associated with higher grain yield; however, they often result in reduced concentrations of essential micronutrients, specifically zinc (Zn) and selenium (Se), while responses for magnesium (Mg), a secondary macronutrient, remain variable. In contrast, organic production systems typically exhibit comparatively lower grain yields but significantly higher micronutrient densities, thereby highlighting their potential contribution to nutritional enhancement.

The inverse relationship between grain yield and micronutrient concentration shown in Figure S5 (Supplementary Materials) reflects the classical dilution effect, whereby increased carbohydrate accumulation under high-input conditions leads to a relative reduction in mineral concentrations within grain tissues [4,49].

Importantly, this trade-off is not absolute and can be alleviated through appropriate genotype selection and optimized nutrient management strategies. For instance, genotype IC8 (a wheat genotype characterized by enhanced selenium accumulation) combined relatively high grain yield with superior Se concentration, whereas genotype P5 (a high-yielding wheat genotype) achieved maximum yield potential with moderate micronutrient levels. These findings suggest the existence of genotypic variation in nutrient partitioning efficiency among wheat cultivars [52].

The physiological basis underlying this trade-off is closely linked to nutrient functions and interactions summarized in Table 2. Macronutrients such as nitrogen (N) and sulfur (S) play a central role in protein synthesis and grain quality formation, whereas phosphorus (P) and potassium (K) regulate energy transfer, enzymatic activity, and osmotic balance, thereby influencing grain filling and yield stability [5,44,53,115,116]. In parallel, micronutrients—including Zn and Se—are essential for enzymatic functions, antioxidant defense mechanisms, and human nutrition, and their accumulation in grains is highly sensitive to both soil availability and plant physiological status [2,40,117].

Agronomic interventions summarized in Table 3 provide practical pathways to mitigate the yield–quality trade-off. Balanced nitrogen:phosphorus:potassium (N:P:K) fertilization, foliar application of zinc (Zn), selenium (Se) biofortification, and the incorporation of organic amendments have all been shown to enhance micronutrient accumulation without substantially compromising yield. Furthermore, INM systems—which combine organic and inorganic nutrient sources—can improve nutrient-use efficiency and soil health, thereby reducing micronutrient dilution under high-yield conditions [36].

From a breeding perspective, the targets outlined in Table 4 emphasize the development of wheat ideotypes with enhanced nutrient-use efficiency and biofortification capacity. Key traits such as NUE, sulfur-use efficiency (SUE), Zn uptake and translocation efficiency, and Se accumulation are governed by complex genetic mechanisms. These traits can be effectively exploited through approaches such as quantitative trait loci (QTL) mapping, marker-assisted selection (MAS), and genomic selection [2,5,34,39].

Future research should prioritize the integration of genotype × environment × management (G × E × M) interactions to identify stable wheat genotypes capable of maintaining both high grain yield and superior nutritional quality across diverse production systems. Moreover, linking physiological traits (Table 2), agronomic strategies (Table 3), and breeding targets (Table 4) within a unified and integrative framework will be essential for designing resilient and nutritionally enhanced wheat production systems. The relationship illustrated in Figure S5 (Supplementary Materials) further underscores the importance of such integrative approaches, as overcoming the yield–quality trade-off remains central to addressing global food security challenges and micronutrient malnutrition.

#### 4.6.2. Integrated Nutrient Management (INM): A Path Forward

**Figure 5:**
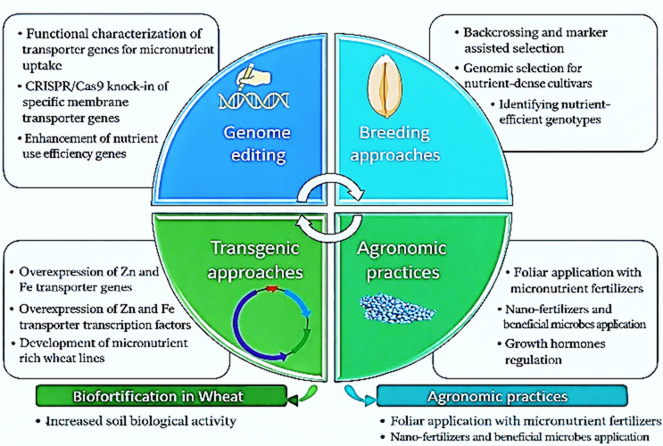
Overview of agronomic, genome editing, transgenic, and breeding approaches to biofortification in wheat [119].

Sustainable wheat intensification requires a paradigm shift toward INM that:

1. Embraces Synergies: Applies N, S, and micronutrients in balanced, synergistic ratios based on quantitative interaction matrices. The N × S synergy is particularly critical for achieving high yields with superior grain quality.
2. Leverages Biofortification: Combines agronomic and genetic tools to enhance grain nutritional density. Foliar Zn-Se applications provide immediate solutions [39], while breeding—accelerated by marker-assisted selection and genomic selection—builds long-term sustainability.
3. Adapts to Systems: Develops tailored management practices and cultivars for organic, conventional, and intermediate systems. IC8’s superior performance under organic conditions and P5’s responsiveness to conventional fertilization demonstrate the importance of system-specific adaptation [91].
4. Builds Resilience: Uses nutrition as a tool to bolster crop resilience against abiotic stresses, particularly through S, Zn, and K management. The K × Si synergy offers a non-nitrogen approach to enhancing heat-stress tolerance [50].
5. Integrates Biotechnology: Employs marker-assisted selection, genomic selection, and CRISPR-mediated gene editing to accelerate development of nutrient-efficient cultivars [109]. The integrated strategies illustrated in the figure demonstrate the complementary roles of genetic, biotechnological, and agronomic approaches in improving micronutrient accumulation in wheat. Genome editing techniques, particularly CRISPR/Cas9-based modifications, enable the functional characterization and targeted manipulation of membrane transporter genes responsible for micronutrient uptake. These approaches enhance nutrient-use efficiency by introducing precise modifications in genes regulating Zn and Fe transport and assimilation (Figure 5).
6. Efficient Input Use: Enhances nutrient-use efficiency through synergistic combinations, with the potential to reduce total nutrient inputs while maintaining yield and decreasing environmental footprint.

Despite the advances and integrated strategies discussed above, several critical knowledge gaps still limit the full realization of these approaches under diverse production systems. Identifying and addressing these gaps is essential to further refine nutrient management frameworks and achieve sustainable improvements in wheat yield and grain nutritional quality.

### 4.7. Research Gaps and Future Directions

**Figure 6:**
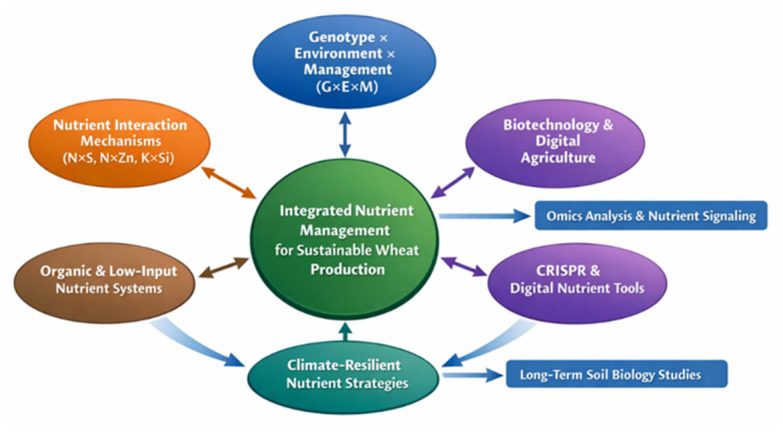
Framework of major research gaps and future research directions in wheat nutrient management and biofortification.

Despite considerable progress in understanding wheat nutrition, biofortification strategies, and INM systems, several important research gaps remain. These gaps limit the ability to fully optimize wheat productivity, grain nutritional quality, and environmental sustainability simultaneously. The major knowledge gaps and emerging research priorities are summarized conceptually in Figure 6, which illustrates the interconnected relationships among nutrient interaction mechanisms, genotype × environment × management integration, organic and low-input production systems, climate-resilient nutrient strategies, and emerging biotechnological and digital agriculture approaches. Addressing these research domains collectively will be essential for advancing sustainable wheat production systems [2,120,121,122].

#### 4.7.1. Limited Understanding of Nutrient Interaction Mechanisms

Although numerous studies have demonstrated important nutrient interactions in wheat systems, the physiological and molecular mechanisms underlying these relationships remain insufficiently understood. Interactions such as nitrogen × sulfur (N × S), nitrogen × zinc (N × Zn), and potassium × silicon (K × Si) play critical roles in regulating plant growth, nutrient uptake efficiency, and grain nutritional composition. For instance, sulfur availability regulates nitrogen assimilation and the synthesis of sulfur-rich amino acids, which directly influence gluten structure and grain protein quality [1,39].

Similarly, recent research indicates that silicon can enhance potassium transporter activity under abiotic stress conditions, thereby improving osmotic regulation and photosynthetic efficiency in wheat plants [50]. Despite these findings, most studies have focused on pairwise nutrient interactions under controlled environments, while real agricultural systems involve complex multi-nutrient networks influenced by soil chemistry, climate variability, and plant genotype.

Future research should integrate omics-based approaches, including transcriptomics, proteomics, and metabolomics, to elucidate nutrient signaling pathways and regulatory networks governing nutrient interactions in wheat [2,120]. As illustrated in Figure 6, understanding these interaction mechanisms represents a foundational step toward developing more efficient INM systems.

#### 4.7.2. Integration of Genotype × Environment × Management (G × E × M) Interactions

Crop performance is determined by the complex interplay among genetic traits, environmental conditions, and agronomic management practices. However, many current studies still examine these components independently. Increasing evidence suggests that wheat genotypes differ substantially in their responses to nutrient availability, fertilization regimes, and environmental conditions [94].

Multi-environment trials demonstrate that some wheat cultivars maintain stable yield and micronutrient accumulation across diverse environments, whereas others show strong environment-specific responses. These genotype × environment interactions can significantly influence nutrient uptake efficiency, grain protein content, and micronutrient accumulation.

As highlighted in Figure 6, integrating G × E × M frameworks into wheat research will be essential for improving nutrient-use efficiency and biofortification outcomes. Future breeding programs should combine genomic selection, phenotypic screening, and agronomic optimization to identify cultivars that perform well under specific nutrient management strategies and environmental conditions [83,91].

#### 4.7.3. Nutrient Management in Organic and Low-Input Systems

While conventional fertilization strategies have been extensively studied, comparatively fewer investigations have focused on nutrient management under organic and low-input agricultural systems. Organic production relies heavily on biological nutrient cycling processes, soil organic matter dynamics, and microbial activity, which influence nutrient availability and plant uptake patterns [121,122].

Several studies have reported that organic systems can enhance micronutrient concentrations in wheat grain, particularly zinc and selenium, due to improved biological activity and reduced chemical competition among nutrients [2,92]. However, yield reductions are often observed due to slower nutrient release and lower nitrogen availability during critical growth stages.

As shown in Figure 6, further research is needed to better understand nutrient cycling processes in organic systems, identify wheat genotypes adapted to low-input environments, and develop fertilization strategies that yield productivity with enhanced grain nutritional quality [85].

#### 4.7.4. Climate-Resilient Nutrient Management Strategies

Climate change represents one of the most significant challenges for future wheat production. Increasing temperatures, water scarcity, and extreme weather events can negatively affect nutrient uptake, translocation, and grain filling processes, ultimately influencing grain yield and nutritional composition [49].

Certain nutrients play important roles in improving plant resilience to abiotic stress. For example, potassium contributes to osmotic regulation and stomatal control, while sulfur and zinc participate in antioxidant defense mechanisms that protect plants from oxidative damage [42]. Silicon has also been shown to enhance plant tolerance to heat and drought stress by improving structural stability and water-use efficiency [50].

The conceptual framework presented in Figure 6 highlights the need for climate-smart nutrient management strategies that integrate soil fertility management, crop genetics, and environmental adaptation. Future research should focus on identifying nutrient combinations that improve crop resilience while maintaining high productivity and grain nutritional quality under changing climatic conditions.

#### 4.7.5. Emerging Biotechnological and Digital Agriculture Approaches

Recent advances in genomics, genome editing, and digital agriculture technologies offer promising opportunities for improving wheat nutrient efficiency and grain nutritional quality. Tools such as genomic selection, marker-assisted breeding, and CRISPR-based gene editing enable targeted manipulation of genes involved in nutrient transport, remobilization, and storage [18,123].

Despite their potential, the practical implementation of these technologies in wheat breeding programs remains limited. Challenges include regulatory barriers, insufficient field validation, and limited integration between genetic innovations and agronomic practices.

As illustrated in Figure 6, combining biotechnological tools with digital nutrient management systems could significantly enhance nutrient-use efficiency. Advances in remote sensing, precision agriculture, and crop simulation modeling now enable real-time monitoring of plant nutrient status and soil variability, allowing more precise fertilizer recommendations [124,125].

Future research should therefore focus on integrating genomic innovations with digital decision-support systems, enabling farmers to optimize fertilizer applications while minimizing environmental impacts and improving grain nutritional quality.

## 5. Conclusions

Sustainable improvement in wheat productivity requires moving beyond conventional single-nutrient approaches toward a more integrative understanding of how nutrient interactions, fertilization strategies, and genetic factors collectively regulate plant performance. This review highlights that wheat growth and nutrient-use efficiency are governed by coordinated nutrient dynamics, with key interactions such as nitrogen and sulfur playing fundamental roles in metabolic regulation and productivity. In parallel, balanced micronutrient management, particularly involving zinc and selenium, emerges as a practical pathway for enhancing nutritional value through biofortification. A central contribution of this work is the establishment of a system-oriented framework that links nutrient interactions with genotype-specific responses and environmental variability, providing a unified perspective for optimizing wheat production. This synthesis further demonstrates that production outcomes are strongly context-dependent, shaped by the interplay among management practices, environmental conditions, and genetic potential. The importance of genetic variability in nutrient-use efficiency and adaptation is emphasized, supporting the integration of modern breeding approaches with fertilization strategies. Emerging technologies, including genomic-assisted selection and gene editing, offer significant opportunities to accelerate the development of nutrient-efficient and biofortified wheat cultivars.

Future research should focus on elucidating the biological mechanisms underlying nutrient interactions, validating integrative approaches under diverse field conditions, and developing decision-support systems that translate scientific insights into practical applications. Additionally, leveraging emerging technologies such as high-throughput phenotyping, advanced genomic selection, and AI-assisted crop modeling can accelerate the development of nutrient-efficient and biofortified wheat cultivars. Expanding the exploration of genetic diversity, breeding for stress-resilient and nutrient-efficient varieties and integrating findings into farmer-oriented management tools will be critical to maximize both productivity and nutritional quality. Overall, adopting a systems-based strategy that integrates plant nutrition, genetics, and environmental management will remain essential for achieving sustainable wheat production with improved yield and nutritional outcomes.

## Figures and Tables

**Table 1:** Effects of Sulfur Fertilization on Wheat Yield and Quality.

Factor	Response Range (%)	Key Conditions	Remarks
Grain Yield	+4 to +18	Low soil sulfur availability (<7.2 mg kg^−1^); no-tillage systems; post-legume rotations	Stronger response under sulfur deficiency; requires balanced N supply; variability linked to soil sulfur status
Grain Protein	+2 to +6	Observed across genotypes; more consistent under balanced fertilization	Moderate but relatively stable improvement; variability depends on sulfur availability
Protein Quality	Improved	Adequate sulfur availability	Enhances glutenin synthesis and dough strength

**Table 2:** Physiological roles of major nutrients affecting wheat yield and quality.

Nutrient	Primary Role	Effect on Yield	Effect on Quality	References
N	Chlorophyll synthesis, protein formation	• ↑ Tiller & spike density • ↑ Grain number	• ↑ Protein (S-dependent)	[34,39,116]
S	Sulfur amino acids, coenzymes	• ↑ N remobilization	• Enhances gluten & dough strength	[5,116]
P	ATP formation, nucleic acids, membranes	• ↑ Biomass • ↑ Spikelet development	• Maintains protein & starch	[116]
K	Osmotic balance, stomatal conductance	• ↑ Grain filling • ↑ Water-use efficiency	• ↑ Protein quality	[49]
Zn	Enzyme cofactor, root growth	• ↑ Nutrient uptake	• ↑ Grain Zn	[2,40]
Se	Antioxidant, stress tolerance	• Maintains yield under stress	• ↑ Grain Se	[117,118]
Mg	Photosynthesis, carbohydrate partitioning	• ↑ Biomass • ↑ Stress tolerance	• Supports protein stability	[49]

↑ indicates an increase.

**Table 3:** Agronomic strategies for improving wheat grain yield and nutritional quality.

Strategy	Target Trait	Production System	References
Balanced N:P:K fertilization	Yield, protein quality	Conventional/Organic	[5,56]
Foliar Zn application	Grain Zn	Conventional/Organic	[40]
Selenium biofortification	Grain Se	All	[91]
Organic amendments/compost	Micronutrient density	Organic	[117]
Integrated nutrient management	Yield stability, nutrition	All	[34,36]

**Table 4:** Breeding targets for nutrient-efficient and biofortified wheat ideotypes.

Trait	Physiological Basis	Breeding Approach	References
NUE	N assimilation & remobilization	Phenotypic selection, QTL mapping	[34,39]
SUE	Protein composition	Phenotypic selection, Genomic selection	[5]
Zn efficiency	Root uptake & translocation	QTL mapping, MAS	[2,40]
Se accumulation	Antioxidant activity	Genotype × environment selection	[91]
Mg stability	Photosynthesis	Genotypic selection	[49]

NUE = Nitrogen-use efficiency; SUE = Sulfur-use efficiency.

## Data Availability

The original contributions presented in the study are included in the article; further inquiries can be directed at the corresponding author.

## Supplementary Materials

The following supporting information can be downloaded at: https://www.mdpi.com/article/10.3390/life16050795/s1, Figure S1. Structural framework showing the main macronutrient components and their functional interactions in wheat nutrient management; Figure S2. Schematic diagram of selenium (Se) uptake, translocation, and biofortification in wheat, highlighting soil sources (selenate and organic matter), fertilization systems, and genotypic variation affecting grain Se accumulation; Figure S3. Polygon view of GGE biplot (which-won-where model) showing 7 elite wheat lines in organic (O) and conventional fertilization (N) environments [78]; Figure S4. Conceptual Nutrient Interaction Matrix of Key Nutrient Pairs in Wheat; Figure S5. Trade-off between grain yield and micronutrient concentration in wheat under organic and conventional fertilization systems; Table S1. Dataset and Reported Response Ranges for Nitrogen and Sulfur Effects on Wheat Yield and Grain Protein.
