# Supplementary material for: Advancing Wheat Productivity Through Nutrient Interactions, Fertilizer Practices, and Genetic Improvement
Source: Life (Basel). 2026 May 10;16(5):795. doi: 10.3390/life16050795 (PMC13208743; doi:10.3390/life16050795)
Supplement: Supplementary file 1 [file life-16-00795-s001.zip › life-4241087-supplementary.pdf]

Supplementary Materials

Title: Supplementary Materials for:

Advancing Wheat Productivity Through Nutrient Interactions, Fertilizer Practices, and Genetic Improvement

Figure S1.

Structural framework showing the main macronutrient components and their functional interactions in wheat nutrient management.

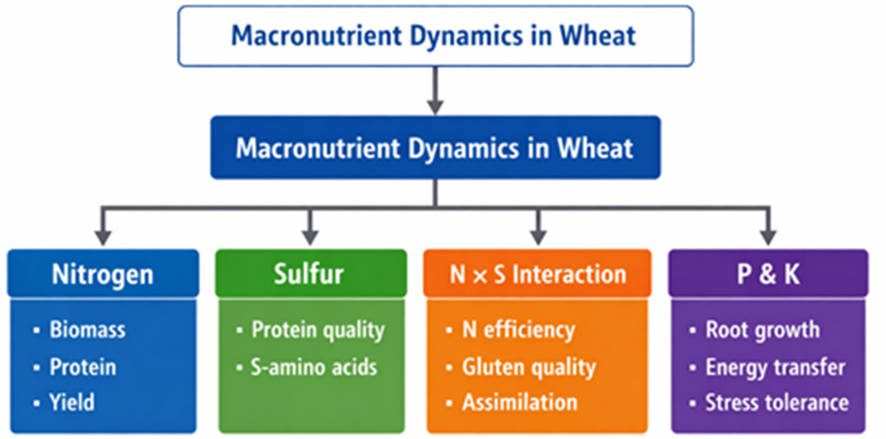

Figure S2.
Schematic diagram of selenium (Se) uptake, translocation, and biofortification in wheat, highlighting soil sources (selenate and organic matter), fertilization systems, and genotypic variation affecting grain Se accumulation.

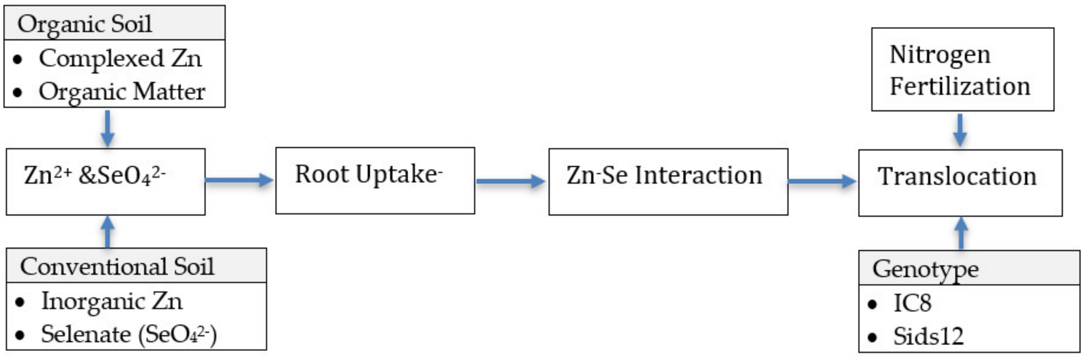

Figure S3.
Polygon view of GGE biplot (which-won-where model) showing 7 elite wheat lines in organic (O) and conventional fertilization (N) environments [78].

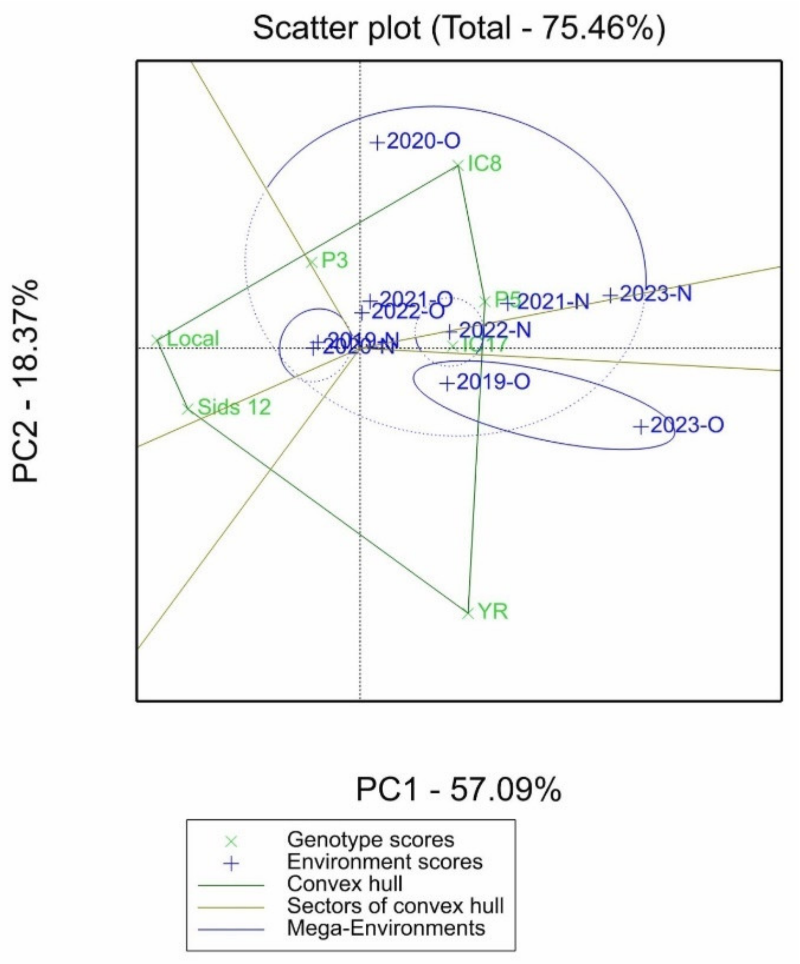

Figure S4.

Conceptual Nutrient Interaction Matrix of Key Nutrient Pairs in Wheat. A schematic heat-map representation illustrating hypothesized synergistic and antagonistic interactions among key macro- and micronutrients (N, P, K, S, Zn, Fe, Ca, and Si). Cell colors represent qualitative interaction strength based on synthesis of published literature rather than quantitative statistical estimation. Diagonal elements (self-interactions) are shown in light grey and are not interpreted.

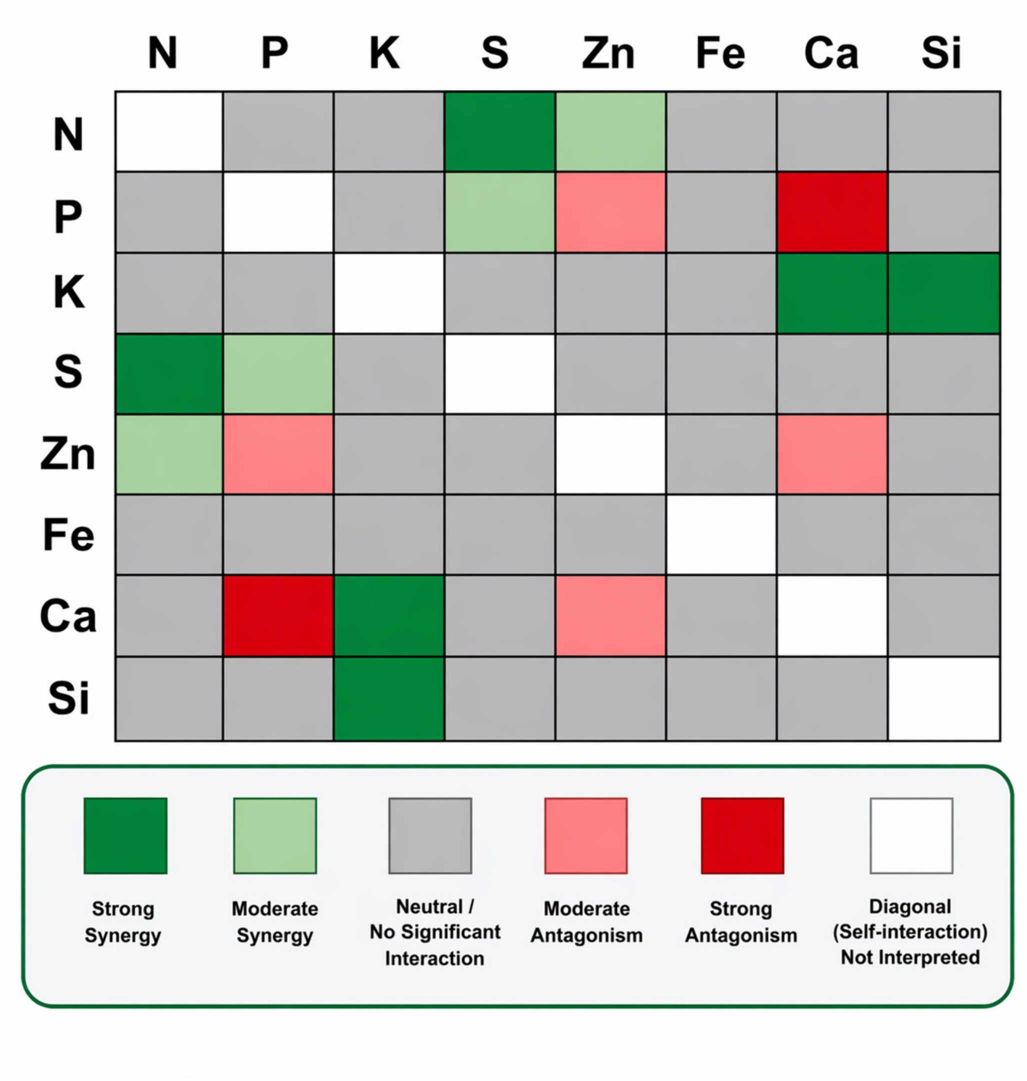

Figure S5.
Trade-off between grain yield and micronutrient concentration in wheat under organic and conventional fertilization systems.

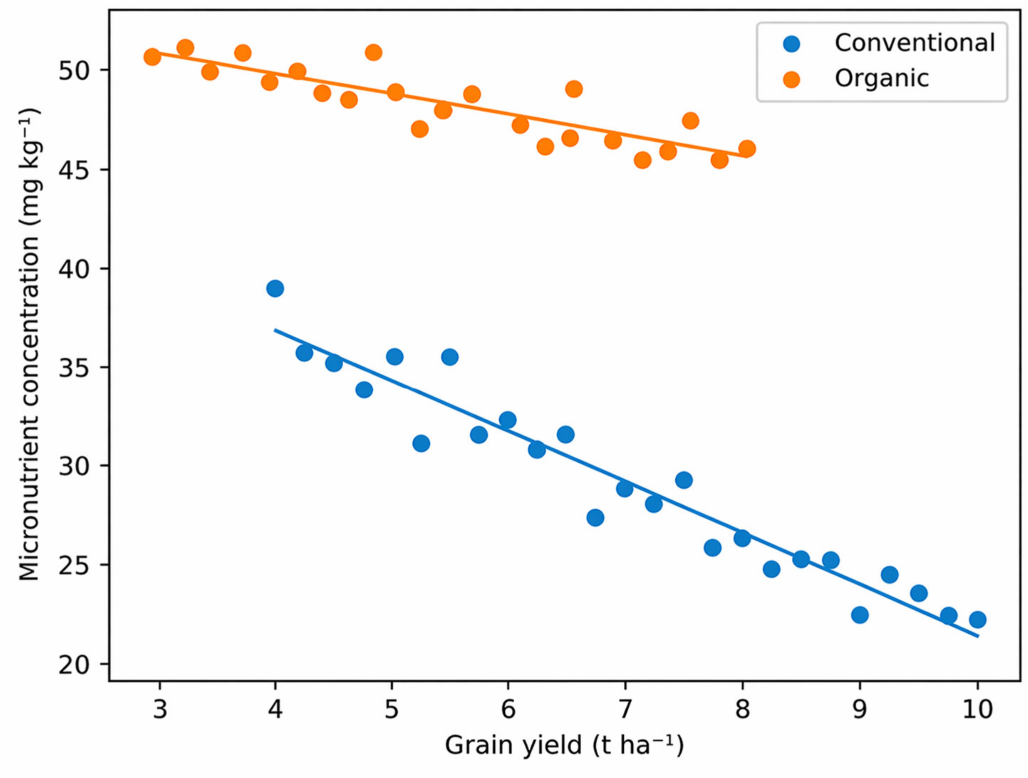

Table S1. Dataset and Reported Response Ranges for Nitrogen and Sulfur Effects on Wheat Yield and Grain Protein

Study ID	Reference	Study Type	Treatment Comparison	Yield Response* (%)	Protein Response (%)	Conditions	Notes
S1	[35]	Meta-analysis	Combined N+S vs N-only/control	12–18	4–6	Stronger under sulfur-deficient conditions	Global synthesis of N–S responses
S2	[47]	Meta-analysis	Split N vs single N	8–12	3–5	Broad environmental conditions	Nitrogen-only benchmark
S3	[43]	Field study	N+S vs low sulfur	10–16	4–6	Sulfur-deficient soils	Protein quality effects
S4	[39]	Review	Combined N+S vs N-only	10–17	4–6	Variable soil conditions	Nutrient use efficiency improvement
S5	[46]	Field study	Nitrogen rate variation	0 to >50 (variable field response)	0–4	Heterogeneous soil and management conditions	Yield–protein trade-off under N limitation
S6	[23]	Meta-analysis + experimental synthesis	Sulfur application vs control	4.2	2.1	Sandy and low organic matter soils	Confirms N–S interdependence

*Response values were compiled from meta-analyses, field experiments, and review studies. Reported values represent observed ranges under diverse agronomic conditions, rather than newly calculated averages. Differences among studies reflect environmental variability, soil properties, and nutrient management practices. Nitrogen-only responses are included as a benchmark to contextualize the contribution of sulfur fertilization.
